# Bringing cultured meat to market: Technical, socio-political, and regulatory challenges in cellular agriculture

**DOI:** 10.1016/j.tifs.2018.04.010

**Published:** 2018-08

**Authors:** Neil Stephens, Lucy Di Silvio, Illtud Dunsford, Marianne Ellis, Abigail Glencross, Alexandra Sexton

**Affiliations:** a Social and Political Sciences, Brunel University London, Kingston Lane, Uxbridge, UB8 3PH, United Kingdom; b Charcutier Ltd, Felin y Glyn Farm, Pontnewydd, Llanelli, SA15 5TL, United Kingdom; c Kings College London, Floor 17, Tower Wing Guy's London, United Kingdom; d Dept of Chemical Engineering, Claverton Down, Bath, BA2 7AY, United Kingdom; e Weston Park Farm, Weston, SG1 7BX, United Kingdom; f Oxford Martin School, University of Oxford, 34 Broad Street, Oxford, OX1 3BD, United Kingdom

**Keywords:** Cellular agriculture, Cultured meat, Clean meat, *In vitro* meat, Society, Regulation

## Abstract

**Background:**

Cultured meat forms part of the emerging field of cellular agriculture. Still an early stage field it seeks to deliver products traditionally made through livestock rearing in novel forms that require no, or significantly reduced, animal involvement. Key examples include cultured meat, milk, egg white and leather. Here, we focus upon cultured meat and its technical, socio-political and regulatory challenges and opportunities.

**Scope and approach:**

The paper reports the thinking of an interdisciplinary team, all of whom have been active in the field for a number of years. It draws heavily upon the published literature, as well as our own professional experience. This includes ongoing laboratory work to produce cultured meat and over seventy interviews with experts in the area conducted in the social science work.

**Key findings and conclusions:**

Cultured meat is a promising, but early stage, technology with key technical challenges including cell source, culture media, mimicking the in-vivo myogenesis environment, animal-derived and synthetic materials, and bioprocessing for commercial-scale production. Analysis of the social context has too readily been reduced to ethics and consumer acceptance, and whilst these are key issues, the importance of the political and institutional forms a cultured meat industry might take must also be recognised, and how ambiguities shape any emergent regulatory system.

## Highlights

• Cellular agriculture includes tissue engineering and fermentation based approaches.
• Five key technical challenges with cultured meat production are made explicit.
• Social issue studies disproportionately focus upon ethics and consumer acceptance.
• New analysis of political, institutional and regulatory issues is required.

## 1 Introduction

Cultured meat involves applying the practices of tissue engineering to the production of muscle for consumption as food. Sometimes also known as clean meat or *in vitro* meat, it is an emergent technology that operates as part of the wider field of cellular agriculture and in a relation of competition and collaboration with innovation in plant-based proteins. This paper contributes to a growing number of review papers about cultured meat (Arshad et al., 2017; Datar & Betti, 2010; Kadim, Mahgoub, Baqir, Faye, & Purchas, 2015; Post, 2012). What is distinct about this paper is a willingness to engage in articulating the practical challenges facing the field and the call for extending the socio-political debate on cultured meat beyond ‘ethics’ and ‘consumer acceptance’ to include complex policy issues like food transitions and practical regulatory mechanisms. Subsequently this paper focuses upon a detailed account of cultured meat. While we recognise the value of conducting a direct comparison between cultured and traditional meat systems or other innovative approaches, such comparative work is beyond the scope of this paper and we direct readers to other contributions for this work (Alexander et al., 2017; Bonny et al., 2015).

While the paper is of international relevance, as the issues described are applicable in all territories, we base our more grounded and detailed discussion on the policy context of pre-Brexit UK as this is the locality we understand best. Our analysis derives from a diverse set of collective experiences as participants and analysts of the cultured meat and livestock meat contexts, and is supported by over seventy expert interviews with participants active in cellular agriculture and other alternative protein developments over a five-year period. Interviewees include professionals engaged in producing cultured meat and other cellular agriculture products within both universities and in companies, funders of this work, and complimentary interviews with professionals working in plant-based meat substitutes and edible insects.

The global livestock industry has come under increasing scrutiny in recent years due to the scale of its environmental, ethical, and human health impacts (Scollan et al., 2011). These concerns, coupled with projections that demand for protein products will continue to rise over the coming decades (Gerber et al., 2013), means there is an urgent need for methods of protein production that are more sustainable, nutritious and animal welfare-conscious. Protein analogues (non-animal proteins) already go some way towards achieving this; however, the desire to eat meat and animal-derived foods has led to the emergence of cellular agriculture, which aims to produce animal proteins using fewer animals and less animal-derived material than the current livestock industry, by utilising culturing techniques. This approach aims to marry a consumer desire to eat meat with the drive to ensure global food security, a nutritious diet, and reduce the environmental burden of food production.

Research has shown a rise in UK consumers incorporating more vegetarian and vegan choices into their diets (Caldwell, 2015). Informa Agribusiness Intelligence estimates that by 2021 UK sales of meat analogues will grow by 25% and milk alternatives by 43%; such growth will take the total UK sales of milk alternatives from £149 million (US$208 million) to £299 million (US$400 million) (FoodBevMedia, 2017). Significant growth is also expected at the global scale, with a recent estimate predicting the global protein analogue market to be worth $46 billion (GBP£33 billion) by 2020 (Business Wire, 2018). Globally, it is projected that the protein analogue market will generate £5.2 billion in revenue by 2020 (Allied Market Research, 2014). Yet despite projections of market and cultural shifts appearing to favour protein innovation, there is a critical need to examine cultured meat within the contexts of existing food policy and the existing material landscape of food production. If the associated technical and consumer-perception challenges that have been identified in other literature, and to which this document speaks to in later sections, can be overcome there is real potential for these technologies to instigate considerable material and regulatory changes to local, national and international food systems. Understanding what these changes are and what consequences – both positive and negative – they may bring is, we argue, an important task to conduct while these technologies remain relatively nascent in their development.

In seeking to address these issues, this article (i) provides an overview of the state-of-the-art of cultured meat technology, (ii) situates it among broader innovations in cellular agriculture, (iii) discusses the potential benefits of cultured meat, (iv) details the technical challenges faced, and (v) identifies key consumer, political and regulatory aspects of cultured meat. These last two sections on technical and social issues also articulate some of the challenges, weaknesses, and concerns about cultured meat technology.

## 2 Cultured meat and cellular agriculture

### 2.1 Cultured meat

In the early 2000s, two projects were conducted to produce cultured tissue for food purposes: one by a NASA-funded college-based group (Benjaminson, Gilchriest, & Lorenz, 2002), and another by a team of bio-artists in the Tissue Culture and Art Project (Catts & Zurr, 2010). Both projects produced small quantities of tissue, with the NASA group performing sniff-tests to assess palatability, and the bio-arts team conducting taste-tests as part of an arts performance piece. In 2005 the Dutch government funded the first of two three-year research projects in the area based upon PhD studentships that sought to culture porcine adult and embryonic stem cells (du Puy, 2010; Wilschut, Jaksani, Van Den Dolder, Haagsman, & Roelen, 2008), develop an algae-based culturing medium (Tuomisto & de Mattos, 2011), and use electronic and chemical stimuli to induce mouse muscle cell growth (Langelaan et al., 2011). From this Dutch work came the field's most high-profile moment when Professor Mark Post of Maastricht University secured financial support from Google co-founder Sergey Brin to produce the world's first cultured beef burger, which was cooked and eaten in a London press conference in August 2013 (O'Riordan, Fotopoulou and Stephens, 2016; Post, 2014). Over the last five years a number of small companies have emerged - some with their own prototypes - although none have yet surpassed the visibility of the 2013 cultured burger.

The technology involves expanding stem cells then differentiating them into muscle cells. This is typically done using chemical/biological cues in the cell culture media (Langelaan et al., 2011) and mechanical stimulation. However, in the field of tissue engineering there is evidence that physical material properties can be used instead, or as well, and we expect developments in scaffolds to be a necessary part of cultured meat research.

Much of the most advanced work in the field is conducted within start-up companies that can be selective in which information about their process they make publically available, and, subsequently, it can be difficult to know exactly what each company is doing. Given this, here we provide an account of current activity to the best of our knowledge. One leading team is Mark Post's Maastricht group that produced the world's first cultured burger with primary bovine skeletal muscle cells, and links Maastricht University and spin-off company Mosa Meats. Another is US-based start-up company Memphis Meats who have produced demonstration cultured products in the form of a meatball, beef fajita, chicken and duck. During 2017, vegan mayonnaise company Just (formally Hampton Creek) announced they would have a cultured meat product on the market during 2018, and have released promotional video footage of cultured chicken nuggets (Just, 2017). In Israel the company ‘Super Meats’ has been operating in connection with the Hebrew University of Jerusalem for several years, and recent news reporting suggests three Israeli cultured meat companies - SuperMeat, Future Meat Technologies, and Meat the Future – will benefit from the $300 million trade deal signed between China and Israel (Roberts, 2017). While this deal has been reported in the press, it is not yet clear publically exactly what this will mean for these companies, and none have yet publically revealed any demonstration products. US-based start-up Modern Meadow also produced demonstration ‘steak chips’ - dehydrated, edible, high-protein food products formed of cultured muscle cells that were combined with a hydrogel (Modern Meadow et al., 2015) – although the company currently focuses upon cultured leather. Another more recently established US start-up, Finless Foods, is working on cultured fish, although they describe their work as early stage. There are a number of small and early stage start-ups, some of which do not at the time of writing have a website, that are known to have entered the field, and some of which have already left. There are also a number of University laboratories with an interest in the field, for example third sector cultured meat advocacy group New Harvest have funded Research Fellows at the University of Bath, University of Ottawa, Tufts University, and North Carolina State University.

While we can provide short accounts of the history and technological approach of cultured meat, it remains a challenge to provide a definitive account of what it is. This is because, as a profoundly novel product and radically different way to produce meat compared to existing livestock methods, its status as a well-defined and widely accepted entity remains contested. In 2010 Stephens argued cultured meat (then called *in vitro* meat) was best described as an “as-yet undefined ontological object”, to capture the way in which this new type of thing, with little in the way of history or precedent, had entered our world to disrupt and sit uncomfortably within the existing ways we categorise and understand what meat is (see also Stephens, 2013). Little framework existed to make sense of this new type of tissue presented as meat outside of science fiction narratives (McHugh, 2010), and many did not know how to rationalise it. As part of the promotional work of the 2013 cultured burger event one definition of cultured meat was given some visibility. In this definition cultured meat is meat as we know it, an identical product just produced “not in a cow” (Post, 2013), and it is to be consumed by people who like meat but are concerned about the environmental and animal welfare impacts of livestock production methods. While this clearly did bring a framework of understanding to cultured meat, it was still a framework that could be contested by some (Laestadius & Caldwell, 2015; Laestadius, 2015; O'Riordan, Fotopoulou & Stephens, 2016), and remains unknown to others.

One alternative definition is provided by Hocquette (2016), who argues cultured meat is most accurately described as “artificial muscle proteins” (p169) because ‘meat’ implies maturation inside an animal and the process of slaughter. Another view would define cultured meat not as a final meat product, but as an ingredient that a meat producer could work into a final meat product. Potentially this could be mixed with other ingredients, including plant-based or traditional animal-based meat ingredients. Alternatively, if potential consumers express what has been termed the ‘yuck’ response, cultured meat could be recognised as simply not fit for consideration as food at all (Van der Weele & Driessen, 2013). The key point at this stage is not to think we can know the ultimate, future definition of cultured meat at this point in time, but to recognise that as a highly novel and distinct artefact the exact status of what it is remains ambiguous, contested, and political, and may continue to be for some time.

This contestation extends to what it should be called, even within the community of people working to produce and support the technology. The early work in the decade following the millennium mostly used the term ‘*in vitro*’ meat. Around 2011 the term cultured meat became used more as the word 'cultured' captured cell culturing techniques, emphasised similarities to fermentation processes such as beer and cheese, and had an appealing resonance as artful and creative (Datar, 2016; Kramer, 2016; Stephens & Lewis, 2017). Since 2015 some within the field, most notably the third-sector group the Good Food Institute, have been advocating the term clean meat, primarily because it is believed to be more appealing to consumers and focuses attention on why it is ‘clean’ as opposed to why it is ‘cultured’, which is thought to have a more positive implication (Friedrich, 2016). Notably, all three names retain ‘meat’, even though it would be imaginable to have a different name, for example Hocquette's (2016) focus upon muscle protein. Of course, outside of the community, there have been a number of derogatory terms used in public debate, including lab meat, synthetic meat, and Frankenstein meat. Terminology is important in framing how things are understood, and this contestation over what it is called reflects both the ambiguity over what it is, and the political sensitivities of how different groups want it to be positioned.

### 2.2 Cellular agriculture

Cellular agriculture encompasses a set of technologies to manufacture products typically obtained from livestock farming, using culturing techniques to manufacture the individual product. There is still debate as to exactly how cellular agriculture should be defined, and which (proposed) products fit within or beyond this definition. However, within the community associated with cellular agriculture there is some agreement that it can be divided into two types that here we term tissue engineering-based and fermentation-based cellular agriculture, grouped by the production method used.

Tissue engineering-based cellular agriculture includes cultured meat and leather systems in which cells or cell lines taken from living animals are tissue engineered in an effort to produce useable tissue with minimal quantities of animal tissue input compared to livestock methods in which the cells themselves form the product. Starting material, i.e. the cells, can be taken from an animal using a biopsy procedure (Post, 2014), or a genetically-modified cell line could be produced that only requires animals from which to source the original cells (Genovese, Domeier, Prakash, Telugu, & Roberts, 2017). Modern Meadow's early leather work also used a tissue engineering approach.

Fermentation-based cellular agriculture contrasts to tissue engineering-based systems in that it does not use any tissue from a living animal. Instead products are manufactured by fermentation using bacteria, algae or yeast that have typically been genetically modified, by adding recombinant DNA, so they produce organic molecules. These molecules can be used to biofabricate familiar animal products (e.g. gelatine, casein (used for milk), and collagen (used for leather)). Fermentation-based cellular agriculture draws upon commonplace industrial biotechnology and therefore may result in marketable products in a shorter timeframe compared to tissue engineering cellular agriculture that relies on technology that has not been proven at large scales. Modern Meadow, who were the first to produce steak chips, now primarily focus upon leather and have shifted away from a tissue engineering approach towards a fermentation-based system in which an undisclosed cell type is genetically instructed to produce a specific type of collagen for manipulation into leather. Other examples include start-ups Clara Foods (egg white), Pembient (rhinoceros horn), and Perfect Day - previously Muufri – (milk).

Datar, Kim, and d’Origny (2016) note an equivalent distinction within the field but based on the cellular content of the product. Their preferred terminology is ‘cellular agriculture products’ for what we term ‘tissue engineering-based cellular agriculture’ and ‘acellular agriculture products’ for what we term ‘fermentation based-cellular agriculture’. In their account cellular agriculture products “are made of living or once-living cells” while acellular agricultural products “contain no cellular or living material” (p128). Here we depart from Datar, Kim and d’Origny's nomenclature because the term acellular suggests a lack of cells which obfuscates the roles of the microbes that are themselves single-cell organisms. Their usage also suggests both cellular and acellular products are subsets of cellular agriculture, which, while potentially accurate, is a confusing mode to describe the distinction made.

A key feature of both forms of cellular agriculture products is the aspiration to produce what we term ‘biologically equivalent’ products to the livestock versions. This can extend to targeting molecularly and genetically identical material that delivers viscerally equivalent eating or usage experiences. It is the goal of biological equivalence that separates cellular agriculture from a new wave of plant-based protein analogue projects including Beyond Meat and Just's egg-like products that also seek ‘viscerally equivalent’ experiences but absolutely avoid biological equivalence. Processed cultured meat products, such as the demonstration products produced by Memphis Meats, aspire to biological equivalence.

For the remainder of this paper we focus upon cultured meat.

## 3 Potential benefits of cultured meat

The benefits of a cultured meat system are articulated more fully in other review articles such as Datar and Betti (2010), Kadim et al. (2015), and Post (2012). Here we summarise the key themes, before addressing the challenges and opportunities of realising these more fully across the rest of the paper, although importantly we note our account includes greater emphasis on potential farming perspectives about the benefits than these other published accounts.

Cultured meat could deliver reduced water use, greenhouse gas emissions, eutrophication potential, and land use compared to conventional livestock meat production. This potential has been assessed in a number of Life Cycle Assessments, although all are based upon hypothetical models of what form cultured meat production might take. Tuomisto et al. (2011) compared cultured meat to conventionally produced beef, sheep, pork and poultry, finding it involves approximately 78–96% less greenhouse gas emissions, 99% less land use, 82–96% less water use, and 7–45% less energy use, depending upon what meat product is it compared to (although poultry uses less energy). Mattick, Landis, Allenby, and Genovese (2015) produced a second comparative study using a different model for cultured meat production, with the most notable differences being the media production method used and inclusion of a cleaning phase. These results suggest cultured meat could involve some trade-offs, with significant energy use leading to cultured meat having greater global warming potential than pork or poultry, but lower than beef, while retaining significant gains in land use. Using a different field of comparison, Smetana, Mathys, Knoch, and Heinz (2015) conducted a cradle-to-plate assessment to compare cultured meat to a range of meat alternatives – plant-based, mycoprotein-based, and dairy-based - and chicken, as the least environmentally problematic conventional meat. Across a set of environmental categories they found that cultured meat had the highest impact, mostly due to its high energy level requirements, with the only exceptions being land use and terrestrial and freshwater ecotoxicity. The overall picture is that cultured meat could have less environmental impact than beef, and possibly pork, but more than chicken and plant-based proteins. However, all three Life Cycle Assessments note that cultured meat technology has significant scope for innovation that could reduce the energy requirements below those used in these assessments, and subsequently could deliver better environmental outcomes than these models predict.

Another potential benefit is that cultured meat could be less prone to biological risk and disease through standardised production methods, and through tailored production could contribute to improved nutrition, health and wellbeing (Post, 2012). However there are some areas to address around genetic instability of multiple cell divisions (Hocquette, 2016) and the media components (Dilworth & McGregor, 2015); while the latter will not be consumed, a full analysis of traceability of components would ensure transparency of the science. Furthermore, being less reliant on climate, land quality and area (FAO, 2013) it has also been proposed that cultured meat could enable more of the global population to have consistent access to protein, although we return to the politics of access later in the paper.

Cultured meat aims to use considerably fewer animals than conventional agriculture. From an animal protection perspective this could appeal to vegans, vegetarians and to those conscientious omnivores interested in reducing their meat intake on ethical grounds (Hopkins & Dacey, 2008).

While the precise economic value of harvested cells has yet to be determined, the potential to harvest large numbers of cells from a small number of donor animals gives rise to the possibility of considerably higher returns per animal than traditional agriculture. This level of profitability could provide a credible alternative to intensive farming systems such as Concentrated Animal Feeding Operations (CAFO).

Cultured meat could also provide new opportunities within traditional agriculture for those utilising traditional native breeds of livestock. The move from carcass to cell harvesting could see a shift change away from the genomic and phenotypic selection of high yielding, hybridised breeds of livestock to the utilisation of more traditional livestock who can thrive on low density, low input, extensive systems. The benefits are three-fold: these low impact systems have a much lower environmental impact, have the potential to be highly profitable, and could potentially contribute to the retention of the genetics of traditional breeds and will safeguard their biodiversity.

When considering food waste, traditional carcass utilisation within the commercial meat industry is the single biggest problem in the context of waste management. Cultured meat provides a new opportunity, whereby the prime cut alone is produced for consumption or processing rather than the whole carcass.

There is also opportunity for each producer to create their own version of the product (much like craft brewers, farmhouse cheesemakers and charcuterie producers now), therefore giving them diversity and competitiveness in the market, as well as engaging in higher skilled jobs in a new knowledge economy. If developed in such a way as to support it, the combination of traditional agriculture and new technologies will enable a circular economy as the majority of waste products (heat, metabolites) from cultured meat production can be upgraded for use on a farm or sold. There is also the opportunity to establish a true cost accounting structure to realise both the financial and environmental impact of the production of food through cellular agriculture.

## 4 Technical challenges of producing cultured meat

The challenge of producing cultured meat is to replicate the muscle-growing environment found in a cow or other animal and recapitulate it in the laboratory or factory. Muscle development has evolved over millions of years and as such it is an efficient process perfectly suited to occurring in the body as part of a vast array of other functions. Tissue engineering of muscle, as for any tissue, combines biological understanding of tissue development and growth, with biochemical engineering principles to replicate the in-vivo environment. To date, tissue engineering has largely been focused on medical applications such as regenerative medicine, and non-animal technologies for *in-vitro* models used for drug discovery and toxicology. The technical principles are the same for producing cultured meat, but for meat the scale is much larger and the product must be affordable as a commodity. This noted, cultured meat is a food rather than a medical product so the regulatory requirements need not be so stringent, and the grade (purity) of raw materials may not need to be as high as biomedical applications.

### 4.1 Meat, muscle and *in-vitro* myocyte culture

To understand the technical challenges, definitions of meat, muscle biology, and *in-vitro* culture of muscle cells need to be considered. The European Union legislative definition of meat is “skeletal muscle with naturally included or adherent fat and connective tissue” (European Parliament, 2003). Structurally, meat is an exsanguinated and dehydrated product of the musculoskeletal system that can be formed of a number of tissues including skeletal muscle, bone, connective tissues, blood vessels and nerves. It is predominantly skeletal muscle which is bound to bone via tendons and connected to each other via a network of connective tissues of varying compositions but predominantly composed of collagen (Gillies & Lieber, 2011). There are three processes by which skeletal muscle is formed: embryonic myogenesis, adult skeletal myogenesis and muscle regeneration after trauma (Grefte, Kuijpers-Jagtman, Torensma, & Von Der Hoff, 2007). *In-vitro* skeletal muscle tissue engineering aims to mimic regeneration of muscle after trauma and/or embryonic myogenesis. Although cell type and maturation pathways may differ, the end goal is to obtain a terminally differentiated cell capable of proliferating and differentiating into muscle fibres.

Generating muscle begins during embryogenesis where the first muscle fibres are formed from mesoderm derived structures. Muscle-resident myogenic progenitor cells then proliferate and continue until a steady state is reached (Bentzinger, Wang, & Rudnicki, 2012). Once the muscle has matured these muscle cells enter a quiescent state and lie between the basal lamina and sarcolema of myofibres (Zammit, Partridge, & Yablonka-Reuveni, 2006). They are known as muscle stem cells (satellite cells) which mature into myocytes, the building blocks of new adult muscle. Upon trauma, muscle repair and regeneration happens in three stages: inflammatory response; activation, proliferation, differentiation and fusion of satellite cells; and the maturation and remodelling of new myofibres (Yin, Price, & Rudnicki, 2013). These stages are not mutually exclusive and coincide. The aim of *in-vitro* culture is to mimic the in-vivo environmental niche in order to create skeletal muscle comparable to native tissue, which would aim to replicate either the embryogenesis or regeneration pathway depending on the starting cell source. Typically, tissue engineering for cultured meat focuses on growing myogenic ‘muscle’ cells (myocytes) alone via the regenerative pathway, as these are the main constituent of meat. However, to achieve muscle tissue that has the potential to fully replicate meat, multiple cell types are required. Here, we focus on myocytes, as these have been considered frequently in the context of cultured meat. The majority of skeletal muscle analysis has been carried out in 2D experiments using cell lines (Burattini, 2004). However, 3D structures (‘bioartificial muscle’) are being investigated in regenerative medicine and as an alternative *in-vitro* model, as a better representation of native skeletal muscle tissue (Snyman, Goetsch, Myburgh, & Niesler, 2013). For cultured meat, thin 3D cultures can be utilised to form processed meats (burgers, sausages) whereas carcase meats would need the optimisation of thicker 3D structures with a nutrient and oxygen supply and waste removal to sustain the inner core of cells.

### 4.2 Tissue engineering of muscle for consumption as cultured meat

The extent to which the biology of muscle is replicated will determine the complexity of the tissue engineering process. A like-for-like piece of muscle (e.g. steak) is the long-term goal. This requires a complex system containing multiple cell types growing in an organised manner, and a structure that will need a replicated blood vessel network. A more simplistic and near-future goal is producing a muscle protein ingredient based on muscle cells alone. Despite these longer term differences, many of the challenges at this point in time are the same for both, and are outlined below.

#### 4.2.1 Cell source

It is currently widely debated as to how best to source the cells. There are two possible cell sources to form tissue engineered cellular agriculture products: primary cells isolated from the original tissue, or cell lines. Cell lines can be formed two ways. One method is typically via induction (genetic engineering or chemical), which can program the cells to proliferate indefinitely (Eva et al., 2014). Another is to select spontaneous mutations where the cell expresses immortality and culture the resulting population (ThermoFisher, 2017). These immortalised cells could decrease the dependency on fresh tissue samples and increase the speed of proliferation and differentiation. However, sub-culturing, passaging, misidentification, and continuous evolution are just some of the problems that can occur using cell lines (National Institutes of Health 2007). Furthermore, it has been argued that these cells are not always representative of the primary cell, they may show different growth rates for example, hence cell data should be interpreted with caution. The conversion of somatic cells into induced pluripotent stem cells (iPSC) are another option, and while this is relatively new technology, promising developments are being made (Genovese et al., 2017; Wu & Hochedlinger, 2011).

The other option is harvesting primary cells found in native tissue, perhaps from a small herd of animals on an intermittent basis, and culturing them. Muscle stem cells (satellite cells) are the most researched source, but other multipotent cells such as mesenchymal stem cells are being studied due to their higher proliferation capacity (Stern-Straeter, 2014) and ability to be expanded using serum-free media (Chase, Lakshmipathy, Solchaga, Rao, & Vemuri, 2010; Jung, Panchalingam, Rosenberg, & Behie, 2012; Oikonomopoulos et al., 2015). Embryonic stem cells, which proliferate indefinitely, are an alternative, however, directing towards a muscle cell lineage is more difficult. There are also human primary cell sources available for research (CookMyoSite, 2016), but these also only give a representation of the myogenic characteristics of a specific species (Sultan & Haagsman, 2001), and for this particular source, the culture of human tissue for meat would have enormous ethical, health, and regulatory implications. Challenges of using primary cells include isolation of the desired cell type from the harvest tissue, both with regard to homogeneity and cell numbers; this can be technically challenging, costly and often result in insufficient numbers of cells for any meaningful data to be acquired. Furthermore, inter-sample variability will impact growth behaviour and response to the culture environment. There is still much debate as to the optimal cells to use in terms of animal type, breed, and tissue from which the cells are taken.

#### 4.2.2 Culture media

The culture media used for both stages of skeletal muscle development is usually supplemented with 10%–20% of growth media (Bian & Bursac, 2009, Hinds, Bian, Dennis, & Bursac, 2011, Mudera, Smith, Brady, & Lewis, 2010, Fujita, Endo, Shimizu, & Nagamori, 2010, Smith, Passey, Greensmith, Mudera, & Lewis, 2012). Foetal calf serum or horse serum is added between the range 0.5–2% at the differentiation stage (Burattini, 2004; Chiron et al., 2012). Chicken embryo extract is also used as an addition to some cultures. Optimisation of the culture media is highly dependent on the cell species origin (Burton, Vierck, Krabbenhoft, Bryne, & Dodson, 2000). In addition, it is common practice to add antibiotic or antibiotic/antimitotics to cells in cultures to prevent infection particularly for long-term cultures.

Serum contains a wide range of growth factors, hormones, vitamins, amino acids, fatty acids, trace elements and extracellular vesicles required for cell growth (Aswad, Jalabert, & Rome, 2016; Brunner et al., 2010). There have been studies utilising serum-free media through the addition of supplementary proteins (Shiozuka & Kimura, 2000) or new branded media such as AIM-V (Fujita et al., 2010), Sericin and Ultroser-G (Fujita et al., 2010; Portiér, Benders, Oosterhof, Veerkamp, & van Kuppevelt, 1999), with promising results. For example, AIM-V has shown increased active tension over serum media during the differentiation stage. Further studies must be conducted to remove serum from the whole culturing process to reduce dependency on animal products. Standard cell culture media contains inorganic and organic components including carbohydrates, amino acids and vitamins required to maintain cell viability in the cultured cell population (Arora, 2013). However, if commercial media are to be used in a product, a Life Cycle Assessment must be conducted for the purposes of cellular agriculture, although this is complicated because in most cases the proprietary nature of commercial media means the source, extraction method and processing of components remains unknown.

#### 4.2.3 Mimicking the in-vivo myogenesis environment

The building blocks of muscle are adherent cells which are immobile and embedded within the tissue. To mimic the natural environment and 3D structure, a scaffold is required with appropriate characteristics to allow cell adhesion and subsequent proliferation and tissue development. An alternative is to develop a cell line that is non-adherent, and is one which would greatly reduce cost and the carbon footprint of the cultured meat production process; however, this is in very early stages of development and will not be covered here. Scaffolds for muscle tissue engineering have been extensively described in the literature (Chan & Leong, 2008; Sakar et al., 2012; Vandenburgh, Karlisch & Farr, 1988). However, it should be noted that successful scaffolds for 3D skeletal muscle formation are all currently animal-derived due to factors such as cell adhesion, fibre alignment and comparability to an in-vivo environment (Bian et al., 2009). The additional consideration is whether the scaffold should be part of the product and therefore edible and degrade during the culture process to leave ‘just’ the cultured meat; or, whether the cells are removed from the scaffold so it can be reused to save material. Cost is also important and it is expected that new scaffolds will continue to be developed for as long as cultured meat products are themselves developed and re-developed.

These systems all present their own array of challenges for tissue engineering-based cellular agriculture. There are numerous considerations, for example, the use of medical grade collagen, fibrin, thrombin or other animal derivatives to produce hydrogels, to mimic the natural tissue. We must also consider the hydrogel constituents being different in composition from the native muscle extracellular matrix; the intricate nature of anchor points causing replication and scalability issues; gel contraction causing cell congregation at the edges of the gel along lines of tension, more so than in the core of the formed fibre (Chen, Nakamoto, Kawazoe, & Chen, 2015); inconsistency in mature fibre production and alignment; limitation in tissue thickness; and, solid scaffold degradation rate, uncoupling from or edibility in the muscle tissue created. Both proliferation of cells and differentiation to specific cell type and tissue must be optimised and scaled. In the case of myogenic cell cultivation inadequate research has been conducted, in particular in relation to differentiation.

To date, the only successful muscle tissue constructs have been a few hundred microns in thickness, which is acceptable for minced but not whole muscle cuts (Lovett, Lee, Edwards, & Kaplan, 2009). Cell sheets are being explored for thicker tissue construction (Hinds, Tyhovych , Sistrunk & Terracio, 2013), however, for highly structured and organised tissues the engineering of highly perfused scaffolds would be required. Investigations have turned to forming channels within the tissue, and there has been specific research into 3D structured tissue formation using channelled networks made from sacrificial scaffolds (Mohanty et al., 2015), removable structures and lithography (Muehleder, Ovsianikov, Zipperle, Redl, & Holnthoner, 2014), whereby flow could be perfused throughout the tissue. 3D-printing seems a promising concept in creating these channelled networks, with examples including cultured leather purveyors Modern Meadow patenting a method and device for ‘scalable extrusion of cultured cells for use in forming three-dimensional tissue structures’, and Harvard researchers 3D-printing a perfusion network that was able to sustain a culture for six weeks (Kolesky, Homan, Skylar-Scott, & Lewis, 2016).

#### 4.2.4 Use of animal-derived and synthetic materials for the scaffold and media

In most cases, both the synthetic scaffolds and culture media used could contain animal-derived products. Cells, not surprisingly, grow best on materials found in the body such as collagen, which is commonly used in cell culture systems as a substrate. Cell culture protocols often utilise other compounds found in the body such as growth factors and blood serum added to cell culture media. In medical research blood serum is typically foetal calf serum, although other animals, and more mature animals can be used. It is possible, but somewhat more difficult to grow cells under serum-free conditions or using serum replacements; however, this is itself an area of research that is yet to produce a comparable and affordable alternative (Butler, 2015). As with some foods for humans and animals, culture media contains components synthesised in yeast and bacteria, as well as crops. Yeast and bacterial production of ingredients is in fact synergistic with cultured meat production and could itself be classed as cellular agriculture, however, the use of crude oil derivatives to produce components is not sustainable in the longer-term. Muscle cell culture media are expensive, in fact prohibitive on the large scale, therefore, the manufacture of a sustainable, animal-free, affordable media is a major challenge. The same challenge applies to scaffold manufacture. There are a number of animal and non-animal derived biomaterials that have been utilised in tissue engineering. Myogenic cells prefer to reside in animal-derived materials as would be expected as these materials more closely mimic their natural physiological niche. The majority of successful bio-artificial muscle has been grown on scaffolds made from collagen (Snyman et al., 2013) and to date achieving tissue contraction with synthetic biomaterials has proven problematic (Bian & Bursac, 2009). Further research needs to be conducted on non-animal derived or food-grade animal product biomaterials for the formation components of meat (e.g. muscle, fat, blood vessels).

#### 4.2.5 Bioprocessing

Affordable production of cultured meat with a low carbon footprint and minimal waste can theoretically be achieved, however, the scale required for making cultured meat a commodity will be the largest ever for tissue engineering. Precedents can be taken from other bioprocessing such as the fungus-derived mycoprotein foodstuff Quorn (Wiebe et al., 2002), and the large-scale culturing of Chinese Hamster Ovary cells (CHO cells) for pharmaceutical manufacturing applications (Xing, Kenty, Li, & Lee, 2009); however, the complex environment needed and the architecture of muscle introduces new challenges. The bioprocess itself can be considered in four parts: the cell expansion; the cell differentiation; the product manufacture; and the waste valorisation. There are then the raw materials and waste products, plus logistics, factory siting, and other associated infrastructure, and the associated Life Cycle Assessment which is essential to understand the carbon footprint of the process. As just alluded to, the difference between cultured meat bioprocessing and the established bioprocesses is the complexity of the environment for both proliferation and differentiation of muscle cells. Mesenchymal stem cell expansion is relatively well established at bench scale ‘ready’ for clinical scale (since the vast majority of tissue engineering to date focuses on cell therapies). Publications demonstrate expansion in bioreactors up to 5 litres, but with current commercially-available technologies there is potential for bioreactors up to 2000 litres (Schnitzler et al., 2016). To put into context the scale of cultured meat production, in the region of 8 × 10^12^ cells are required to acquire 1 kg of protein from muscle cells, which would need a ‘traditional’ stirred tank bioreactor in the order of 5000 litres. While this volume is commonplace in established bioprocessing it is as yet unproven in tissue engineering and mesenchymal stem cell expansion. Other bioreactor configurations are available that can, in theory, achieve higher cell densities, including fluidised bed bioreactors and hollow fibre membrane bioreactors, but are considerably less established for cell expansion at this point in time. The scale-up (in a few large bioreactors) or the scale-out (in many smaller bioreactors) are key challenges here. The expansion challenge at this scale is likely to be more easily overcome than the differentiation, in the authors' opinion. The achievement of muscle cell differentiation has been reported for *in-vitro* models (Sharples et al., 2012; Smith et al., 2012) and for Post's first burger (Post, 2014), all of which use scale-out methods but only produced a single piece of tissue-engineered model. The case for this scale-out approach is achievable but highly labour intensive and costly, so establishing a scaffold and bioreactor conditions that enable differentiation in larger bioreactors is the major challenge to make cultured meat a commodity.

## 5 Consumer, political and regulatory aspects of cultured meat

### 5.1 Ethical and consumer perspectives on cultured meat

So far the dominant framings of the social issues related to cultured meat have been ethics and consumer acceptance (see, for example Hocquette, 2015i and the special issue it introduces). These remain important issues that require sustained attention. However, we argue that these alone are insufficiently broad to facilitate the necessary consideration of the politics of cultured meat that will allow both the maximisation of potential social benefit, and the fullest articulation of legitimate concerns and hurdles about the technology. In the subsequent section we begin the work of broadening the analytical scope, but first we briefly review key themes in the existing ethics and consumer response/acceptance literature.

#### 5.1.1 Cultured meat ethics

The academic ethics literature generally reports supportive arguments for cultured meat, especially when adopting a philosophically-orientated approach (Dilworth & McGregor, 2015). These accounts are typically based upon the environmental and animal welfare benefits of a successful cultured meat system. Armaza-Armaza and Armaza-Galdos argue developing cultured meat “would be a moral duty” (2010, p518) while Hopkins and Dacey suggest it “might be our moral obligation” (2008 p579). Supportive but less emphatic is Pluhar (2010) who argues that from both utilitarian and rights-based viewpoints we should support cultured meat, although vegetarianism may be a superior moral response. Schaefer and Savulescu (2014) argue cultured meat development is permissible and worth promoting, especially from vegetarian perspectives. Chauvet (2018) argues animal dignity is not violated by producing cultured meat, and Van der Weele (2010) suggests we should invest in cultured meat to at least see if the benefits can be realised, although she notes they may not.

This given, a minority of writers using different perspectives adopt negative positions. Cole and Morgan (2013) argue from a critical animal studies perspective that cultured meat continues the existing fetishisation of meat, and due to its expense could result in a non-meat eating elite who operate guilt free at the expense of the less well-off. Weisberg writes with the critical theory of Marcuse and Ellul that “[u]ltimately, looking to biotechnology to solve ethical crises is fraught with danger and should be avoided” (2015, p52), a position close to Metcalf's (2013) argument that cultured meat is a dangerous example of the decontextualisation and molecularisation of sustainability, and Lee's (2018) ecofeminist perspective of caution towards the emancipatory potential.

#### 5.1.2 Consumer acceptance and public perception studies

A second important area of study has been the opinions of various publics about cultured meat. Sometimes those in the field reduce this to the issue of ‘consumer acceptance’, although we urge the need for a wider framing of this issue beyond likely purchasing decisions to also include broader personal and political convictions, uncertainties, and ambivalences about the societal impact of cultured meat. Importantly this should inform innovation pathways in a form of ‘upstream engagement’ that embeds critical reflection upon novel technologies into their development (Wilsdon & Willis, 2004). Existing studies on perceptions of cultured meat vary in methodology but demonstrate some commonality in finding diverse opinions from the very supportive to the very negative, with many shades of uncertainty in between. Studies of social media and comments on news articles about cultured meat find the perceived unnaturalness of cultured meat can be a problem (Laestadius, 2015; Laestadius & Caldwell, 2015), noting social media can be a key site of resistance (O'Riordan et al., 2015) (there have also been studies of the media reporting itself, with Goodwin and Shoulders (2013) arguing coverage disproportionately draws upon cultured meat proponents, while Hopkins (2015) argues the media over-represents the importance of vegetarian and vegan viewpoints). The diversity of public opinions on cultured meat was also found in a survey of 1890 scientists and students that used multiple correspondence analysis to identify three clear clusters of respondees: those in favour, those against, and those of no opinion (Hocquette, 2015ii). Another online survey of 673 participants based in the US reported this ambiguity in a different way, noting that, while nearly two thirds of respondents said they would try cultured meat, only one third would eat it regularly (Wilks & Phillips, 2017). Focus group studies have been published from the Netherlands (Van der Weele & Driessen, 2013), Finland (Vinnari & Tapio, 2009), the UK (Bows et al., 2012; O'Keefe, McLachlan, Gough, Mander & Bows-Larkin, 2016), Ireland (Department of Agriculture, Food and the Marine, 2013), the US (Hart Research Associates, 2017) and a comparative study of Belgium, the UK and Portugal (Marcu et al., 2014; Verbeke et al., 2015). Most studies report a diversity of responses spanning positive and negative, although the Finnish study found notably lower levels of support for cultured meat, while the Dutch study suggested the more participants learnt about cultured meat the more they were willing to support it. Studying the impact of new knowledge on perception was the key focus of another Dutch study, this time using psychological experiments with 506 responses, which found different stimuli information altered individuals' considered opinions of cultured meat, although it did not affect their instinctive positive or negative response (Bekker, Fischer, Tobi, & van Trijp, 2017) (see Bryant & Barnett, 2018 for a full review of consumer acceptance studies).

While we support the conduct of ongoing studies on the opinions of various publics like those reported here, we also concur with Bekker's finding that novel information impacts perception, and we further stress the opportunities for flux and change in the real-world context of cultured meat that would inevitably shape what information is available to publics in their reckoning about the technology. For example, should cultured meat enter the market it may be after other cellular agriculture products, such as milk or egg white, which may have swifter pathways to wider-scale commercialisation. Subsequently publics' perceptions of cultured meat could be swayed by these earlier experiences if the category of cellular agriculture remains sufficiently robust to continue to draw them together. Even if not, the novelty, pace of innovation, and ambiguities over the status of cultured meat mean a changing context is likely, so while empirical research with publics today remains vital we must remain cautious of their predictive capacity for opinions in future years in which the context may be different. To be clear, these existing studies of consumer acceptance and public perception remain informative and we believe more are needed, but we also stress the need to recognise the potential for perceptions to change.

### 5.2 Social, political and institutional impacts

While these ethical and consumer acceptance issues remain crucial avenues of enquiry, our argument here is that it is equally vital to extend the analysis of cultured meat to also consider the related social, political and institutional implications it may incur. These issues inform each other, and it is vital they are inspected collectively. Numerous narratives in favour of cultured meat and other alternative proteins have emphasised the ability for these novel foods to ‘disrupt’, and thus overcome, the negative impacts associated with conventional livestock production. However, cultured meat has to date existed predominantly in promissory narratives rather than in tangible, material forms (Jönsson, 2016; Stephens & Ruivenkamp, 2016; Stephens, King, & Lyall, 2018). The abundance of this aspiration rhetoric (fuelled largely by corporate and media actors) coupled with the relative lack of scientific assessments, such as Life Cycle Assessments, has made for an ambiguous and at-times prematurely optimistic discourse around cultured meat. It is not yet certain what a cultured meat sector may look like (e.g. few large-scale vs. many smaller-scale producers), nor what inputs will be required (e.g. animal vs. synthetic growth media) and what their respective environmental and ethical footprints will be.

There is consequently much need for continued assessment of the diverse range of impacts that may come with cultured meat as it develops, both positive and negative, and how these may contribute to or reconfigure existing political economies in the global food system (see also Mouat & Prince, 2018). Broad-based engagement on these impacts is needed across a diverse range of academic, practitioner and policy experts working at the coalface of environmental, health, food security, and animal welfare issues. In particular, such analysis should consider who may potentially be the ‘winners’ and ‘losers’ of a cultured meat sector as it emerges. Pluhar (2010, p. 464) states that for cultured meat to be realised as an ethically acceptable solution it would need to be accessible as a consumable product for “people from all economic backgrounds and cultures … if that is their wish”. We argue that similar attention on social and economic equality is also required at the production level. Key questions include: who will produce cultured meat (i.e. farmers, agribusinesses, bioscientists), and more specifically, who is already enabled to adopt, and potentially profit from, this technology; where will production take place (i.e. Global North/South, on farms/in factories); and, what are the associated social, political, environmental and ethical implications of these developments? Concerns have been raised in public focus groups that cultured meat will provide a new frontier for multinational corporations to accumulate further capital and power over the food system (Driessen & Korthals, 2012), a point also raised by Hocquette (2016) who argues it may further support the domination of Global North economies over those of the South. Conversely, others have envisaged the potential for a shift towards localised and more connected relationships with meat production – for example, the ‘pig in the backyard’ scenario discussed by Van der Weele and Driessen (2013) or ideas of community donor herds that live out their lives serving local areas with their slaughter-free cells. Exploring how cultured meat will become situated within existing socio-political relations regarding the commodification of nature (Birch, Levidow, & Papaioannou, 2010), the different scales and geographies of food production and consumption, and the politics of sustainable and healthy eating (Sexton, 2016), is of critical importance for understanding the ability of cultured meat to realise the promises its proponents currently claim. Importantly, this is a task that must be conducted in the current early stages of the technology and as it develops over the coming years.

When considering these potential future relations it is vital to identify and interrogate their underlying assumptions. As just one example, it is clear that some of the narratives on the potential benefits of cultured meat implicitly assume a ‘substitution effect’, implying that rising consumption of cultured meat would equate to declining consumption of conventional meat. This is particularly so for the environmental narratives and the animal welfare narratives that are premised upon reductions in animal suffering and environmental impact through significant reduction in global livestock populations. Under a full substitution effect, all traditional meat production would be replaced by cultured meat leading to dramatic falls in animal-related emissions, land use, and slaughters. However this assumption is as yet unsubstantiated and we could instead consider as a thought experiment the impact of an ‘addition effect’, in which cultured meat production works not to reduce conventional meat production, but instead to increase the total global meat consumption (of cultured and conventional combined). Under a full addition effect, traditional meat production would not decrease at all, and neither would its environmental impacts nor the numbers of animals slaughtered. The potential for addition, as opposed to substitution, is an under-considered aspect of this work. Core to these environmental and animal suffering narratives is the concern that rising populations and incomes mean demand for meat will outstrip global supply. In this circumstance of insufficient supply and significantly increased demand, it seems reasonable at least to consider that conventional meat production might not fall dramatically, especially if cultured meat products were considered less desirable. Such a thought experiment would lead us to consider how an addition effect could be avoided, and what the conditions of future adoption might be. However, the point here is not to argue that the substitution effect would not occur, but instead to suggest we require a more complex engagement with the political aspects of delivering cultured meat, and an ongoing questioning of underlying assumptions within existing accounts (see also Dilworth & McGregor, 2015).

In deciding the policy landscape in relation to cultured meat, we also argue that consideration must be given to how a cultured meat workforce will materialise and the role of governments in providing financial (e.g. subsidies, grants) and training support for smaller-scale producers who wish to transition to cultured meat production. We anticipate the need for a workforce with a range of skills and knowledge levels that extend beyond more traditional roles of, for example, agriculturalists and veterinarians to also include chemists, cell biologists, material scientists, chemical engineers, skeletal muscle scientists, technicians, meat scientists and food technologists. Furthermore, in the event of changes to existing regulations – such as the legal categories of ‘meat’, ‘dairy’ and ‘eggs’ – there is critical need to examine how such changes might affect conventional, non-cellular agriculture businesses.

### 5.3 Anticipated regulatory pathways

A small but important set of literatures on the regulation of cultured meat exists, with Schneider (2013) considering regulation in the United States and Petetin (2014) considering the European Union. Both argue the regulations at the time of writing were inadequate to appropriately deal with cultured meat technology without significant development. In the US case, Schneider (2013) argues the frequently used regulatory model of ‘substantial equivalence’ between cultured meat and livestock meat is inappropriate because, he argues, livestock meat is not a natural version of cultured meat. He then argues the appropriate regulatory pathway depends upon the techniques used in production, suggesting that explant systems (that expand existing animal muscle tissues, (cf Benjaminson et al., 2002) follow Food and Drug Administration (FDA) New Animal Drugs Applications requirements, while scaffold-based production systems that expand from cells as opposed to fully formed muscle tissue (cf Post, 2014), should follow FDA food additive provisions.

In the EU case, Petetin (2014) argues cultured meat would be subject to novel food regulation, but notes that it does not easily fit the framework at the time of writing. Petetin speculates on the benefits of draft 2013 proposals, of which a version was subsequently approved as Official Journal of the European Union (2015), that remove the consideration of substantial equivalence issues that existed in previous EU and current FDA regulation (Schneider, 2013). These new regulations prioritise the precautionary principle via a European Food Safety Authority (EFSA) risk assessment. While we do not have the legal expertise of Petetin in assessing these regulations, we do note a possible error in her analysis in that Petetin's account assumes that cultured meat is not a genetically-modified product; yet as we describe earlier in this review, the potential for genetically modifying the cells is a key issue of contestation within the field with several laboratories pursuing this route. The relevance of this point is that the new EU 2015 Novel Food regulations exclude genetically-modified foods from their remit, instead pointing towards the regulation on genetically-modified food and feed specifically designed for this type of product (Official Journal of the European Union, 2003, 2015). In effect this provides another example of Schneider's observation that different production methods imply different regulatory pathways, and that currently uncertainties around both the technology and the regulation mean identifying a clear pathway remains a task of dealing with ambiguity.

This given, there are key regulatory issues that require attention that are raised here to provoke further discussion, using the pre-Brexit UK context as an example regulatory system. We agree with Petetin (2014) that EU Novel Food Regulation is the most likely pathway, in the UK mediated by the Food Standards Agency (FSA). A key issue will be establishing if cultured meat is a product of animal origin. We believe it likely will be, although challenging this it is worth remembering that (i) when culturing begins the animal cells are a small proportion of material used compared with the culturing media (which may or may not be animal-based), and (ii) cell lines may be considered a processed product. However, assuming cultured meat is understood as of animal origin then regulation in practice would involve a range of organisations. Extracting muscle biopsies and the keeping of a donor herd would likely include Livestock, Animal Welfare & Slaughter Regulation, including the Department for Environment, Food & Rural Affairs, The Animal and Plant Health Agency, the FSA, and Local Authorities. Cultured meat products themselves would require food regulation via the FSA, Local Authority Environmental Health Department, and Local Authority Trading Standards. In both cases local authorities are heavily involved, so due to its complexity we advocate a ‘primary authority’ model in which one local authority with expertise in the area acts on behalf of all other authorities.

A key concern of the regulations will be safety. This requires an awareness of auditing that should be addressed from the outset of animal cell-based cellular agriculture product development as it brings together cell culture and meat science. Here we provide some examples for consideration, although this is not an exhaustive list. For example, in terms of cell sourcing, donation, procurement and testing of human tissues and cells (Commission Directive 2006/17/EC) and Human Tissue (Quality and Safety for Human Application) Regulations (Legislation.gov.uk, 2007) are the only current directives to base donor cell criteria on. Being medically-orientated documents, these would have to be revised for cultured meat to make it economically viable, and recognise that the end product is not human, no longer living, and ingested, and thus requires different and appropriate regulations. However, the principles can be the basis of testing. Cells may be taken from live animals so the Animal Welfare Act (2006) will be of importance. In terms of processing, auditing should include (i) identification of key possible pathogens, and safety measures to inhibit contamination (through a HACCP-based system), (ii) ensuring ageing of meat is greater than 24h to allow for total cell death, (iii) monitoring and quality assurance of cellular functions at each stage (viability, self-renewal, death and differentiation) are pivotal to quality, function and sustainability, assays for cell potency, and testing of genetic stability (Kirouac and Zandstra, 2008), (iv) the managing of metabolic waste by disposal, recycling or upgrading, and (v) production plant hazard and operability study (HAZOP).

Further research will also be needed to confirm or dispel uncertainties over various potential safety issues. Candidate topics for research include the safety of ingesting genetically-modified cell lines, as these lines exhibit the characteristics of a cancerous cell which include overgrowth of cells not attributed to the original characteristics of a population of cultured primary cells (Ruddon, 2003). Ambiguity stems from a lack of, and conflicting research with some work confirming the transfer of DNA, such as Netherwood et al. (2004) and Spisak et al. (2013). The FDA continue to review regulation and standards for food from genetically-modified animals (FDA, 2017).

Safety and auditing methods also link to production facility regulation. This broader category includes issues such as whether large-scale bioreactors are considered agricultural facilities, with UK agriculture subject to significantly more permissive planning requirements if on designated land. Production facilities may also need to be located in high power zones, and be subject to relevant regulation on energy and environment. Furthermore waste removal strategies require attention, for example if cultured meat waste products are considered animal by-products then the Animal Health and Plant Agency may need consulting. Finally, consideration is needed of whether cellular agriculture facilities of different scales require different regulatory pathways, as happens in the UK for other forms of food production.

Another regulatory issue is the potential for food fraud, as evidenced in the 2013 European horsemeat scandal. In the case of cultured meat this falls into two main forms: (i) attempts to pass cultured meat as conventional livestock meat, and (ii) attempts to pass conventional livestock meat as cultured meat. In the case of combined cultured and conventional livestock meat products there could also be the risk of mislabelling the proportions of meat type. In the alcohol industry trackers are becoming commonplace to prove the provenance of a product. A ‘protein tracker’ for cultured meat could provide inspectors with a tool for product verification. In a cultured meat context there is a need for regulatory bodies to be aware of this, and for researchers to develop a set of protein trackers that are suited to cultured meat.

The final regulatory issue raised here relates to cellular agriculture from non-livestock species (including human). A regulatory response will be needed for cellular agriculture products using non-agricultural animals. Categories for consideration include: endangered and protected animals, dangerous animals, companion animals, and importantly, human cellular agriculture. This work should take note of the full range of production scales from industrial through to DIY home-based culturing. Ethical, social, and safety considerations of the permissibility and necessary protections for these forms of cellular agriculture are required.

## 6 Conclusion

This article has aimed to review the current context of cultured meat by focusing upon the interrelated technical and social aspects of the field, while retaining a willingness to critically engage with the technology. We close the paper by looking ahead in anticipation of what may come in the near and further future.

It seems reasonable to argue that the production of small-scale cultured meat products of edible quality should be achievable in the near future and in some regards is possible now. These are likely to be versions of processed meat products that seek visceral equivalence to familiar products. However, the timeline for delivering this at a price competitive point similar to existing processed meat products is less determinate. Large-scale production is significantly more challenging, the key issue being the production of effective and appropriately priced culture media. The most ambitious production target - producing cultured meat on a scale that could make marked impacts on global climate change - is likely to take many decades, if it is at all possible.

The meanings and terminologies associated with cultured meat remain in flux, and this could remain the case for some time. Within the cultured meat field the general consensus is that cultured meat is meat as we know it, although made through other means and designed with altruistic or social and environmental benefits, and that the preferred term is ‘cultured meat' or ‘clean meat’ (although exceptions exist). However, meaning is produced collectively, and while the field may assert this way of thinking, it is not given that societies more broadly will necessarily take these meanings on board, or that these names will not be replaced by others. Meaning production is diffuse, complex, and multifaceted, and the understandings attached to cultured meat could be reshaped by many factors that are beyond the control of the field itself.

We have argued that too many current accounts of the social impact of cultured meat rely on an overly simplistic argument that frames the issue as one of consumer acceptance. If cultured meat technology does reach the scale proposed by some that enables it to deliver meaningful climate change mitigation then this could be part of a significant and potentially global-scale shift in livelihoods, practices and supply chains across multiple sectors beyond just agriculture (e.g. steel and transport). The success of a cultured meat sector would also depend upon complex social apparatus and government policies, including regulation and tax and subsidy regimes. As such, the continued growth of this sector will likely bring considerable social, political and economic implications for multiple and various stakeholders. There is much need therefore for continued critical analysis of these factors to more fully understand who will be impacted and in what ways, and we anticipate seeing these debates, and the range of engaged stakeholders, expand in the coming years.

Research within the field is currently conducted in both University and private start-up environments (although sometimes the two are linked). From publically available information it seems the start-ups are currently more successful in attracting funding through venture capital than the Universities are through government and charity funding streams. At the time of writing there is much optimism within the field as increasing numbers of start-up companies secure funding, often from high-profile sources, through cycles of high expectations and investment. However, there is no guarantee that this cycle will continue to be successful in the longer term. The current investment cycle is fuelled within a context of fast-paced innovation, as novel small-scale prototype and demonstration products are developed and given media and investor attention. However, these relatively quick-wins may dry up as the more intractable challenge of delivering the upscale necessary for lower prices and large-scale distribution slows visible progress, and the current initial burst of investor interest shifts. As is the case in many industries populated by start-ups, it is likely some of the current set of companies will fail, and possible the sector as a whole may experience a collapse as seen in some tech-bubbles. It is important that the field has an inbuilt resilience to retain expertise and support should the investment momentum drop. Key groups in securing this future could be the start-ups that survive any collapse (perhaps through being bought by larger companies), third sector groups such as New Harvest and the Good Food Institute (although these are also susceptible to financial instability), and the Universities.

It is also worthwhile to remain mindful of the possibility that we could see a situation in which we have an economically-viable cultured meat sector that does not deliver all of the more altruistic or social and environmental benefits currently associated with the technology. For example, net global reductions in greenhouse gases or animal slaughters may not be delivered if livestock meat production is not reduced as cultured meat production increases. Furthermore, gains in health or energy use may not be delivered if the organisations producing cultured meat prioritise other factors in their system. It is clear that the current set of cultured meat groups are motivated by altruistic or social and environmental goals and work to develop innovative approaches that maximise potential benefit. However there is no guarantee that these motivations will be shared and pursued by future cultured meat producers, and we are not yet convinced that the benefits are necessarily inherently embedded within the technology, as could be argued. A situation in which cultured meat was economically viable but delivered few of the altruistic or social and environmental benefits would be a disappointment; as such, we urge the field and its stakeholders to remain attendant to supporting the delivery of the projected benefits.

We do accept that cultured meat could still be an important technology for addressing a range of environmental and food security issues. However, we warn against perspectives that position cultured meat as the defining solution. The contemporary context of planetary tipping points, as well as rising global demand for animal-derived foods, clearly presents significant challenges to existing meat production practices; however, care must be taken to recognise the systemic nature of these challenges and that technofix approaches, such as cultured meat, should not be viewed as the only solution. We argue instead for a multi-faceted response which includes a range of approaches, including promoting meat reduction and plant-based proteins, improved waste management strategies, and policy reforms that redress the systemic inequalities within contemporary protein and livestock food systems.

Cultured meat remains an early stage technology with a diverse range of potential benefits and a wide set of challenges. In this article we have reviewed key issues with a preparedness to critically engage with these technical, social and regulatory challenges, highlight uncertainties, and suggest issues for further consideration. As this review demonstrates, there is a valuable literature emerging considering the multiple facets of the challenging and, to some, unusual technologies of cellular agriculture. However, we recognise the need for further research and analysis from a wider set of disciplinary academic and stakeholder positions, working together in interdisciplinary teams to address the technical, social and regulatory challenges. Through a continuing emphasis on interdisciplinary critical engagement with cellular agriculture and its ramifications, a more nuanced set of understandings will emerge leading to more robust socio-technical responses to these challenges and opportunities.
